# Changes in ovarian cancer survival during the 20 years before the era of targeted therapy

**DOI:** 10.1186/s12885-018-4498-z

**Published:** 2018-05-29

**Authors:** Jung-Yun Lee, Sunghoon Kim, Young Tae Kim, Myong Cheol Lim, Boram Lee, Kyu-Won Jung, Jae Weon Kim, Sang-Yoon Park, Young-Joo Won

**Affiliations:** 1 Department of Obstetrics and Gynecology, Institute of Women’s Life Medical Science, Yonsei University College of Medicine, Seoul, South Korea; 2 Gynecologic Cancer Branch & Center for Uterine Cancer, National Cancer Center, Goyang, South Korea; 3 Cancer Registration and Statistics Branch, National Cancer Center, Goyang, South Korea; 4 Department of Obstetrics and Gynecology, Seoul National University College of medicine, Seoul, South Korea

**Keywords:** Ovarian cancer, Survival, Histology, Korea, Chemotherapy, Surgery

## Abstract

**Background:**

The survival of patients with ovarian cancer has improved because of surgery and chemotherapy. This study aimed to estimate the changes in survival rates among Korean women with ovarian cancer prior to the introduction of targeted therapy for ovarian cancer.

**Methods:**

Data were obtained from the Korea Central Cancer Registry regarding patients who were diagnosed with epithelial ovarian cancer between 1995 and 2014. The relative survival rates were calculated for 5-year periods using the Ederer II method. Cox proportional hazard models were created to assess the associations of demographic and clinicopathological factors with ovarian cancer survival.

**Results:**

During the study period, 22,880 women were diagnosed with epithelial ovarian cancer. The 5-year relative survival rate improved from 57.2% during 1995–1999 to 63.8% during 2010–2014 (*P* < 0.001). Survival outcomes improved between 1995 and 1999 and 2010–2014 for the serous and endometrioid carcinoma subtypes (*P* < 0.001). However, no improvements were observed for the mucinous and clear cell carcinoma subtypes (*P* = 0.189 and *P* = 0.293, respectively). Multivariate analysis revealed that younger age, early stage, recent diagnosis, primary surgical treatment, and non-serous histological subtype were favorable prognostic factors.

**Conclusion:**

Survival outcomes have improved for serous and endometrioid epithelial ovarian cancer in the last 20 years. However, no improvement was observed for patients with mucinous and clear cell carcinoma subtypes.

## Background

Ovarian cancer is the most common cause of gynecological cancer-related death in Korea, and causes approximately 1021 deaths annually [1]. The incidence and mortality of ovarian cancer have increased continuously, and 2413 new cases were detected in 2014 [1–3]. Approximately 75% of the newly diagnosed patients have advanced-stage disease, which partly explains the high mortality rate for this cancer [4, 5].

During the last 20 years, there has been an improvement in survival of patients with ovarian cancer [1, 4–6]. A number of strategies have been evaluated with the goal of improving survival, and some of these strategies have become standard treatments for ovarian cancer. For example, debulking surgery has been emphasized because optimal cytoreduction is one of the most significant predictors of survival [7], and previous studies have revealed that optimal surgical cytoreduction improves survival in cases of advanced-stage disease [8]. In addition, paclitaxel plus cisplatin has been introduced as a front-line therapy for ovarian cancer, and provides better survival outcomes than cyclophosphamide-based regimens [9]. After then, platinum-based chemotherapy has been improved with less toxic and equivalent analogs, carboplatin [10, 11], and paclitaxel plus carboplatin is the most commonly used first-line therapy for ovarian cancer. Moreover, better survival rates have been observed in patients with recurrent disease, with a number of chemotherapies having activity even in platinum-resistant settings. Although recent phase III trials have supported the introduction of targeted agents [12–14], their economic cost, limited insurance coverage, and low patient preference have limited the use of these agents in routine clinical practice [15, 16]. In Korea, the addition of bevacizumab to standard chemotherapy was approved in 2013, and the national insurer only began covering the cost of bevacizumab for platinum-resistant recurrent ovarian cancer in August 2015. Therefore, the present study aimed to investigate the changes in the survival rates among Korean patients with ovarian cancer during the last 20 years, and to identify unmet clinical needs that might be targeted to improve outcomes.

## Methods

This study utilized data from the Korean National Cancer Incidence Database (KNCIDB), which includes data from the Korea Central Cancer Registry (KCCR) and information regarding patients’ demographic characteristics, primary cancer site, morphology, diagnosis date, and initial treatment. KCCR was launched as a nationwide hospital-based cancer registry in 1980 by the Ministry of Health and Welfare, and subsequently expanded to cover the entire population in 1999. The present study evaluated survival data from the KNCIDB. The ovary cancer cases were classified according to the International Classification of Diseases for Oncology, 3rd edition [17] and converted according to the International Statistical Classification of Diseases and Related Health Problems, 10th edition (ICD-10: C-56) [18]. We included only cases of epithelial ovarian cancer, diagnosed between 1995 and 2014. All cases of non-epithelial ovarian cancer (e.g. sex-cord stromal tumors and germ cell tumors) were excluded. All cases followed until 31 December 2015.

The present study’s retrospective design was approved by the institutional review board of the National Cancer Center (NCC2017–0168).

Age at the diagnosis was classified as < 40 years old, 40–59 years old, and > 59 years old. Histological subtypes were categorized as serous carcinoma, mucinous carcinoma, endometrioid carcinoma, clear cell carcinoma, and others. Staging information was based on the Surveillance, Epidemiology, and End Results (SEER) summary staging [19], which categorizes cancer spread from its origin (localized, regional, and distant), because the KCCR has collected this information since 2005. Primary treatments within 4 months were categorized as surgery, chemotherapy, and others.

For the survival analyses, we obtained the data from KNCIDB and the mortality data from Statistics Korea. Relative survival is the ratio of the observed survival rate among patients with cancer, compared to the expected survival rate among age- and sex-matched individuals from the general population. We calculated the relative survival rates (RSRs) using the Ederer II method [20]. Furthermore, we divided the patients into 5-year cohorts based on their diagnosis date to evaluate their 5-year RSRs (1995–1999, 2000–2004, 2005–2009, and 2010–2014). The Cox regression proportional hazard model adjusted to estimate hazard ratio (HR) for the age at diagnosis, SEER stage, year of diagnosis, primary treatment (with or without surgery), and histological subtype [21]. The proportionality of hazards assumption over time was tested for each factor [22]. All analyses were performed using SAS software (version 9.3; SAS Institute, Cary, NC, USA).

## Results

**Fig. 1:**
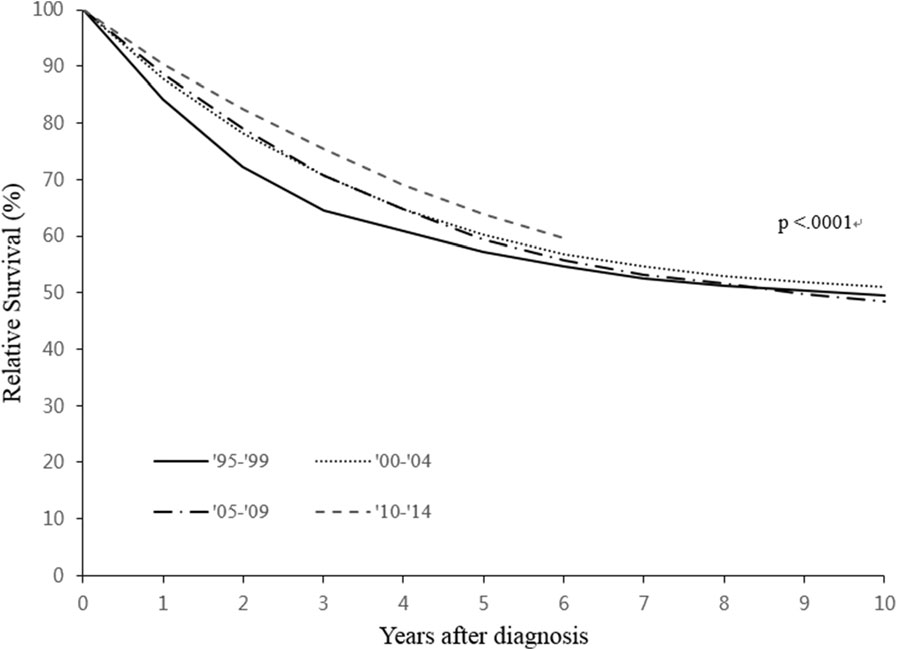
Relative survival rate of ovary cancer by time period

**Fig. 2:**
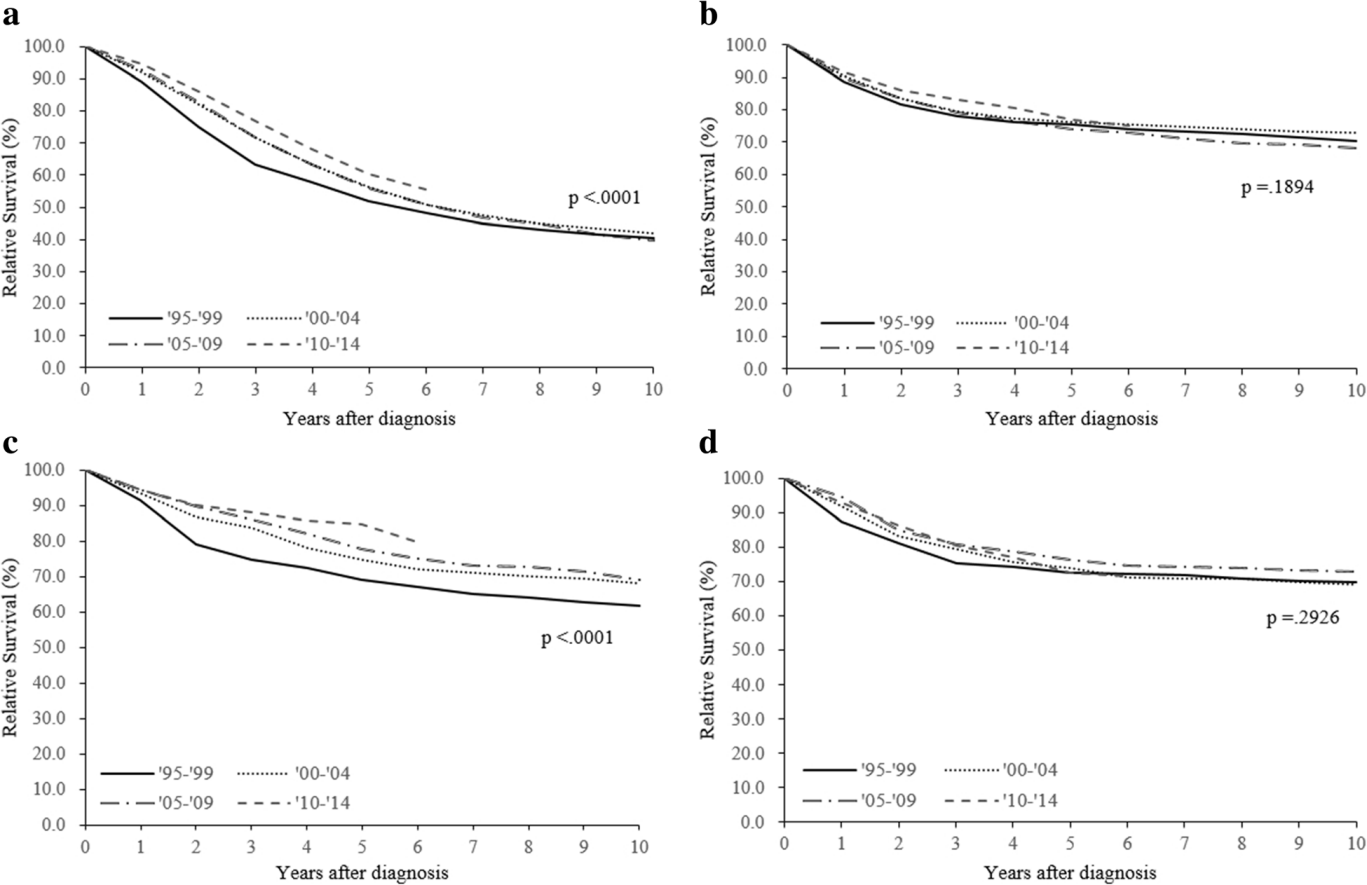
Trends in relative survival rate according to histology and the time period (**a**) serous carcinoma (**b**) mucinous carcinoma (**c**) endometrioid carcinoma (**d**) clear cell carcinoma

A total of 22,880 women were diagnosed with ovarian cancer between 1995 and 2014, and their characteristics are shown in Table 1. The overall 5-year RSR significantly improved during study period (57.2% during 1995–1999, 60.2% during 2000–2004, 59.4% during 2005–2009, 63.8% during 2010–2014; P for trend < 0.001) (Fig. 1). Figure 2 shows the survival outcomes according to histological subtype, which improved for the serous and endometrioid carcinoma subtypes between 1995 and 1999 and 2010–2014 (P for trend < 0.001). However, no significant improvements were observed for the mucinous and clear cell carcinoma subtypes (P for trend = 0.189 and 0.293, respectively).

**Table 1:** Basic characteristics according to the time period of ovarian cancer diagnosis

	Total			1995–1999		2000–2004		2005–2009		2010–2014		
	(*n* = 22,880)			(*n* = 3740)		(*n* = 4863)		(*n* = 6317)		(*n* = 7960)		
	No. of cases	%	*p-value*	No. of cases	%	No. of cases	%	No. of cases	%	No. of cases	%	*p-value*
Age (years)			<.0001									<.0001
< 40	3849	16.8		945	25.3	910	18.7	990	15.7	1004	12.6	
40–59	12,169	53.2		1813	48.5	2526	51.9	3378	53.5	4452	55.9	
> 59	6862	30.0		982	26.3	1427	29.3	1949	30.9	2504	31.5	
SEER Stage			<.0001									<.0001
Localized	3865	16.9		–	–	–	–	1683	26.6	2182	27.4	
Regional	2564	11.2		–	–	–	–	1053	16.7	1511	19.0	
Distant	6795	29.7		–	–	–	–	2843	45.0	3952	49.6	
Unspecified	9656	42.2		–	–	–	–	738	11.7	315	4.0	
Primary treatment			<.0001									<.0001
Surgery only	6007	26.3		1213	32.4	1252	25.7	1626	25.7	1916	24.1	
Chemotherapy only	1680	7.3		326	8.7	371	7.6	414	6.6	569	7.1	
Surgery + Chemotherapy	13,262	58.0		1745	46.7	2713	55.8	3805	60.2	4999	62.8	
Others	1931	8.4		456	12.2	527	10.8	472	7.5	476	6.0	
Histology			<.0001									<.0001
Serous carcinoma	10,837	47.4		1459	39.0	2119	43.6	3186	50.4	4073	51.2	
Mucinous carcinoma	4005	17.5		916	24.5	1027	21.1	951	15.1	1111	14.0	
Endometrioid carcinoma	2191	9.6		399	10.7	494	10.2	580	9.2	718	9.0	
Clear cell carcinoma	1923	8.4		164	4.4	327	6.7	551	8.7	881	11.1	
Others	3924	17.2		802	21.4	896	18.4	1049	16.6	1177	14.8	

*SEER* Surveillance, Epidemiology, and End Results

Table 2 shows the 5-year RSRs of patients with ovarian cancer according to histological subtype and SEER stage. The overall 5-year RSRs improved from 59.4% during 2005–2009 to 63.8% during 2010–2014 (*P* < 0.001). Improved survivals were also observed for early-stage serous carcinoma (from 77.7% during 2005–2009 to 84.1% during 2010–2014). Furthermore, there was a significant increase in the 5-year RSR for advanced-stage serous carcinoma, from 44.1% during 2005–2009 to 49.5% during 2010–2014. However, women with non-serous carcinoma subtypes did not experience a survival improvement, with the exception of women with early-stage endometrioid carcinoma.

**Table 2:** Five-year relative survival rate, by SEER stage and histologic subtype

	2005–2009 (*n* = 6317)	2010–2014 (*n* = 7960)	*p-value*
Early stage^a^	81.5	86.3	<.0001
Serous carcinoma	77.7	84.1	<.0001
Mucinous carcinoma	87.6	88.6	0.379
Endometrioid carcinoma	88.5	93.6	0.047
Clear cell carcinoma	86.4	87.4	0.673
Distant stage	38.7	43.9	<.0001
Serous carcinoma	44.1	49.5	<.0001
Mucinous carcinoma	30.2	31.5	0.247
Endometrioid carcinoma	50.3	60.1	0.752
Clear cell carcinoma	38.0	22.5	0.012

*SEER* Surveillance, Epidemiology, and End Results

^a^Early stage: local + regional

Table 3 shows the results for the age-based changes in the 5-year RSRs. During 2005–2009 and 2010–2014, patients who were 40–59 years old and > 59 years old experienced an increased 5-year survival rate, although younger patients did not experience a survival improvement, regardless of their cancer stage. Patients who underwent surgery had a significantly higher 5-year RSR, compared to patients who did not undergo surgery, and this association strengthened over time.

**Table 3:** Five-year relative survival rate, by age and primary treatment

	2005–2009 (*n* = 6317)	2010–2014 (*n* = 7960)	*p-value*
Age			
Early stage^a^	81.5	86.3	<.0001
< 40	89.1	91.6	0.203
40–59	83.9	88.1	<.0001
> 59	66.8	77.0	0.001
Distant stage	38.7	43.9	<.0001
< 40	46.2	44.7	0.808
40–59	44.9	50.5	<.0001
> 59	28.2	34.1	0.002
Surgery			
Early stage^a^	81.5	86.3	<.0001
with surgery	82.6	87.1	<.0001
without surgery	62.3	60.7	0.383
Distant stage	38.7	43.9	<.0001
with surgery	42.5	47.6	<.0001
without surgery	24.0	27.2	0.413

^a^Early stage: local + regional

In the Cox multivariate model, the significant prognostic factors were age at diagnosis, SEER stage, primary treatment, and histological subtype. Furthermore, year of diagnosis was an independent prognostic factor, with patients who were diagnosed during 2010–2014 being 27% less likely to die, compared to patients who were diagnosed during 1995–1999 (hazard ratio: 0.73; 95% confidence interval: 0.65–0.81) (Table 4).

**Table 4:** Estimated hazard ratio of ovarian cancer prior to the era of targeted therapy

	*N*	No. of deaths	Adjusted HR^b^	95% CI	*p-value*
Age (years)					
< 40	3849	988	ref.	ref.	–
40–59	12,169	4992	1.71	(1.60–1.84)	<.0001
> 59	6862	4328	3.00	(2.79–3.22)	<.0001
SEER Stage					
Early stage^a^	6429	1113	ref.	ref.	–
Distant stage	6795	3777	3.23	(3.02–3.46)	<.0001
Year of diagnosis					
1995–1999	3740	2230	ref.	ref.	–
2000–2004	4863	2671	0.89	(0.84–0.95)	0.000
2005–2009	6317	3227	0.94	(0.85–1.03)	0.185
2010–2014	7960	2180	0.73	(0.65–0.81)	<.0001
Primary treatment					
With surgery	19,776	8122	ref.	ref.	–
Without surgery	1772	1233	1.49	(1.39–1.58)	<.0001
Histology					
Serous carcinoma	10,837	5325	ref.	ref.	–
Mucinous carcinoma	4005	1226	0.71	(0.67–0.76)	<.0001
Endometrioid carcinoma	2191	670	0.69	(0.63–0.75)	<.0001
Clear cell carcinoma	1923	507	0.80	(0.73–0.88)	<.0001
Others	3924	2580	1.49	(1.42–1.57)	<.0001

^a^Early stage: local + regional, HR, hazard ratio; ref., reference; CI, confidence interval; SEER, Surveillance, Epidemiology, and End Results

^b^adjusted for Age, SEER stage, Year of Diagnosis, Primary treatment and Histology

## Discussion

Between 1995 and 2014, there has been a gradual increase in the survival of Korean patients with ovarian cancer. Among women with serous carcinoma, the risk of death from ovarian cancer during 2010–2014 was 4.7% lower, compared to during 2005–2009, and 8.5% lower compared to during 1995–1999. Improvement of survival was found for both early stage and distant stage. However, no improvements were observed for patients with the mucinous and clear cell carcinoma subtypes.

The current approach to managing ovarian cancer involves cytoreductive surgery followed by chemotherapy, and a decrease in the proportion of patients without definitive treatment has been observed during the last 20 years (from 12.2% during 1995–1999 to 6.0% during 2010–2014). Thus, an increasing number of Korean patients have benefited from surgery and chemotherapy, and adherence to the standard treatment guidelines is an independent predictor of improved survival [23, 24]. Furthermore, in the present study, the multivariate analysis revealed that surgery was independently associated with better outcomes.

The use of platinum-based chemotherapy has improved with the development of less toxic analogs (carboplatin), as well as research regarding the optimal dose, schedule, sequence, and duration of treatment. In this context, the Gynecologic Oncology Group (GOG) 0111 and OV10 studies revealed that cisplatin plus paclitaxel was superior to cisplatin plus cyclophosphamide [9, 25]. In addition, the GOG 0158 and Arbeitsgemeinschaft Gynäkologische Onkologie Ovarian Cancer Study Group (AGO-OVAR) studies demonstrated that carboplatin plus paclitaxel was not inferior to cisplatin plus paclitaxel [10, 11]. Thus, the incorporation of paclitaxel into first-line therapy has improved the ovarian cancer survival rate. This change was adopted by Korean gynecologic oncologists during 2000–2004, and may partially explain the improvement in survival between 1995 and 1999 and 2010–2014.

However, after the incorporation of paclitaxel into first-line chemotherapy, the first-line chemotherapy options have not substantially changed during the last decade. Although a randomized phase III trial revealed a survival benefit after treatment using intraperitoneal chemotherapy [26], this procedure has not been widely accepted in Korea. The Japanese Gynecologic Oncology Group (JGOG) 3016 study also revealed the superiority of dose-dense weekly paclitaxel plus carboplatin, compared to the standard dosing of paclitaxel [27], although this approach also has limited acceptance in Korea.

Previous studies have emphasized the importance of debulking surgery for ovarian cancer. Bristow et al. found that maximal cytoreduction was one of the most powerful determinants of survival among patients with advanced disease during the platinum era [8]. Thus, many Korean gynecologic oncologists adopted radical surgery and a multidisciplinary approach that includes general surgeons, thoracic surgeons, and urologists. This approach might also explain the improvement in survival between 2005 and 2009 and 2010–2014, and could highlight the importance of surgery in the era of chemotherapy using paclitaxel plus carboplatin.

Furthermore, advances in chemotherapy for the recurrent and supportive care settings might help improve survival outcomes [6]. During the next decades, enormous changes in survival are expected based on the incorporation of targeted treatments for ovarian cancer. For example, the combination of bevacizumab plus paclitaxel and carboplatin provides a survival benefit in patients with advanced-stage ovarian cancer. In addition, the GOG 218 and International Collaboration on Ovarian Neoplasms trial 7 (ICON 7) studies revealed a progression-free survival benefit in the first-line setting [13, 28], while three randomized phase III trials revealed a survival benefit the recurrent setting [12, 14, 29]. Moreover, mature data from phase II and III trials with PARP inhibitors will be available in the next few years, and Study 19 has already revealed a remarkable survival benefit after olaparib treatment for patients with a *BRCA* mutation and platinum-sensitive recurrence [30]. Based on these results, the Korean Food and Drug Administration approved bevacizumab in 2013 and olaparib in 2016. Nevertheless, targeted drugs were rarely used during the present study’s period, and only a few patients would have received targeted drugs in clinical trials.

Despite the progress in treating serous carcinoma during the last 20 years, we did not observe any survival improvements for the mucinous and clear cell carcinoma subtypes. Although epithelial ovarian cancer has significant heterogeneity and the histological subtype is a well-known prognostic factor, the current management strategies do not consider the histological subtype. Previous studies have confirmed that patients with mucinous tumors have inferior long-term survival, compared to the serous or endometrioid subtypes, which is related to a poor response to platinum-based chemotherapy [31, 32]. However, advances in the pathological diagnosis of ovarian mucinous carcinoma have allowed pathologists to distinguish between primary and metastatic mucinous carcinoma, which has led pathologist to suggest that primary ovarian mucinous tumors are rare [33]. The GOG 241 study aimed to compare the efficacy of carboplatin plus paclitaxel +/− bevacizumab to that of oxaliplatin plus capecitabine +/− bevacizumab as first-line chemotherapy for patients with mucinous adenocarcinoma, although there has been limited enrollment in that study because of this subtype’s rarity.

The incidence of clear cell carcinoma has increased markedly in Korea across all age groups since 1999 [2]. Previous reports have confirmed that women with endometriosis have an elevated risk of developing clear cell carcinoma, and this trend is expected to continue in the near future, based on the increasing incidence of endometriosis in Korea [34, 35]. The JGOG 3017 study compared the efficacy of irinotecan plus cisplatin versus paclitaxel plus carboplatin as a first-line chemotherapy [36], although no subtype-specific survival benefits were observed for the irinotecan plus cisplatin regimen. Chan et al. did not report any change in survival rates for patients with clear cell carcinoma after analyzing the data available on the Surveillance Epidemiology and End Results Database [4]. Therefore, treatment using existing anticancer agents has limited ability to improve the prognosis of patients with clear cell carcinoma. The present study also revealed poor survival outcomes and no improvement in the outcomes for advanced-stage clear carcinoma.

The present study is one of the largest population-based studies to evaluate the survival rate of ovarian cancer using available histological and cancer stage data. Although the present study’s findings are strengthened by the large nationally representative sample of Korean women, there are also several limitations. First, the KCCR database does not include disease information such as International Federation of Gynecology and Obstetrics (FIGO) staging and survival information such as recurrence and the cause of death. Hence, we could not identify the specific cause of death for each case. In addition, the sociodemographic information such as region, residence and hospital cannot be obtained from the KCCR database for research purpose. Therefore, we could not analyze the data obtained for the indicators related to the health system. Second, there is no detailed information regarding the surgery and chemotherapy, such as surgeon specialty, extent of debulking, residual disease, neoadjuvant or postoperative chemotherapy, and the specific regimens. Thus, as we observed an improved survival rate among patients who underwent surgery, it is possible that this finding was biased by the selection of healthier patients in the surgery group. Third, central pathology reviews are not performed for patients who are registered in the KCCR.

## Conclusion

Ovarian cancer survival has improved in Korea during the last 20 years. However, no improvements were observed for the mucinous and clear cell carcinoma subtypes. Given the low survival rate in cases with advanced-stage mucinous/clear cell subtypes, clinical trials with novel treatment strategies are urgently needed to improve clinical outcomes in these cases.

## Abbreviations

FIGO: International Federation of Gynecology and Obstetrics
GOG: Gynecologic Oncology Group
ICD-10: International Statistical Classification of Diseases and Related Health Problems, 10th edition
KCCR: Korea Central Cancer Registry
KNCIDB: Korean National Cancer Incidence Database
RER: Relative excess risks
RSR: Relative survival rate
SEER: Surveillance, Epidemiology, and End Results
